# Effect of vaccination on severity and infectiousness of measles during an outbreak in the Netherlands, 2013–2014

**DOI:** 10.1017/S0950268820000692

**Published:** 2020-03-23

**Authors:** A.S.G. van Dam, T. Woudenberg, H.E. de Melker, J. Wallinga, S.J.M. Hahné

**Affiliations:** 1 Department of infectious diseases, GGD Hart voor Brabant, ‘s-Hertogenbosch, The Netherlands; 2 National Institute for Public Health and the Environment (RIVM), Bilthoven, The Netherlands; 3 European Programme for Intervention Epidemiology Training (EPIET), European Centre for Disease Prevention and Control (ECDC), Stockholm, Sweden; 4 Leiden University Medical Center, Leiden, The Netherlands

**Keywords:** Disease outbreaks, measles, measles-mumps-rubella vaccine, vaccination, infectious disease transmission

## Abstract

An outbreak of measles in the Netherlands in 2013–2014 provided an opportunity to assess the effect of MMR vaccination on severity and infectiousness of measles.

Measles is notifiable in the Netherlands. We used information on vaccination, hospitalisation, complications, and most likely source(s) of infection from cases notified during the outbreak. When a case was indicated as a likely source for at least one other notified case, we defined it as infectious. We estimated the age-adjusted effect of vaccination on severity and infectiousness with logistic regression.

Of 2676 notified cases, 2539 (94.9%) were unvaccinated, 121 (4.5%) were once-vaccinated and 16 (0.6%) were at least twice-vaccinated; 328 (12.3%) cases were reported to have complications and 172 (6.4%) cases were hospitalised. Measles in twice-vaccinated cases led less often to complications and/or hospitalisation than measles in unvaccinated cases (0% and 14.5%, respectively, aOR 0.1 (95% CI 0–0.89), *P* = 0.03). Of unvaccinated, once-vaccinated and twice-vaccinated cases, respectively, 194 (7.6%), seven (5.1%) and 0 (0%) were infectious. These differences were not statistically significant (*P* > 0.05).

Our findings suggest a protective effect of vaccination on the occurrence of complications and/or hospitalisation as a result of measles and support the WHO recommendation of a two-dose MMR vaccination schedule.

## Background

Measles is a highly contagious viral disease. The number of secondary cases from one patient in a fully susceptible population ranges between 12 and 18 [1]. Globally, measles remains one of the leading causes of death in young children, despite the availability of safe and effective vaccines [2]. Initial symptoms of measles, including high fever, cough, coryza and conjunctivitis, develop 10–12 days after exposure. A few days later, a rash develops which usually spreads over the entire body. Complications of measles include pneumonia, otitis media, diarrhoea and encephalitis.

Measles virus infection in vaccinated individuals can be due to primary or secondary vaccine failure. Primary vaccine failure is the failure to respond to the vaccine and occurs in about 5% of one-dose recipients [3]. Secondary vaccine failure is defined as susceptibility due to waning immunity after seroconversion and depends mainly on the time since vaccination and the number of doses received [4]. The relevance of vaccine failure for measles control depends on its frequency of occurrence and the severity and infectiousness of measles in vaccinated individuals. Occasional measles transmission has been reported from twice-vaccinated cases [5]. Limited information available suggests, however, that measles in fully vaccinated individuals is less infectious and presents with milder symptoms than measles in unvaccinated individuals [6, 7].

Vaccination against measles has been part of the Dutch national immunisation programme since 1976. Children are offered vaccination against measles, mumps and rubella (MMR) in a two-dose schedule, at 14 months and 9 years of age. Despite high overall vaccination coverage, large measles outbreaks occurred in 1987–1988, 1999–2000 and 2013–2014, mostly affecting unvaccinated individuals of orthodox Protestant denomination [8, 9]. This group of orthodox Protestant individuals live in a socio-geographically clustered area in the Netherlands, described as the ‘bible belt’. About 40% of this group refuses vaccination because of religious reasons [10]. In the 2013–2014 outbreak, 2700 measles cases were notified predominantly among unvaccinated primary school-aged children of orthodox Protestant denomination. The circulating genotype was D8 [11].

This outbreak provided an opportunity to assess the effect of vaccination on the occurrence of complications, hospitalisation and infectiousness of measles.

## Methods

Measles is a notifiable disease in the Netherlands. We included all notified cases with day of rash onset between 23 May 2013 and 11 March 2014 in our analyses. Confirmed and probable cases are notifiable. A confirmed case was defined as any person not recently vaccinated (to exclude possible MMR induced measles cases) and meeting the clinical and laboratory criteria for measles. The clinical criteria included fever, maculopapular-rash and at least one of the following: cough, coryza or conjunctivitis. The laboratory criteria included either detection of measles-specific IgM antibodies in blood specimens or specific detection of measles virus RNA by polymerase chain reaction (PCR) in throat swabs, oral fluid or urine specimens. A probable case was defined as any person meeting the clinical criteria who has been in contact (<3 weeks prior to the date of onset) with a confirmed case. Regional and national laboratories tested and genotyped all collected specimens.

Clinicians and laboratories reported cases to the Municipal Health Services (MHS). The MHS collected information on the cases by interviewing them or their physician using a standardised measles surveillance form. The MHS notified cases meeting the case definition criteria to the national surveillance database ‘Osiris’.

The standardised surveillance form included a question on vaccination status, which was verified in the national vaccination register, by a vaccination card or by consulting the cases' GPs. Questions on the presence of complications and hospitalisation were other items in the form. Encephalitis, pneumonia, otitis media were defined as complications in the form, next to an open text field for other complications. At the start of the outbreak, questions about the source of infection were added to the standardised surveillance form. MHSs were asked to indicate one or more likely sources for each notified case by recording the unique notification identifier of this/these source(s). A likely source of a case was defined as another notified confirmed or probable case with whom there was contact 7–21 days before the onset of rash and whereby the generation interval of the linked cases was between 9 and 14 days [12]. RIVM separately collected information on the duration of hospitalisation.

We considered two outcomes in our analyses: severity and infectiousness. We defined severity as the presence of at least one complication and/or hospitalisation due to measles. Infectiousness was defined as a case being indicated as a likely source of infection to other cases.

Vaccination status was the independent variable of interest. We excluded cases with unknown vaccination status and those where the vaccination status was not verified by the national vaccination register, by a vaccination card or by a GP. In the analyses of complications, we excluded cases for which no information was available on the occurrence of complications.

We used logistic regression to compare the frequency of complications and infectiousness between unvaccinated, once and at least twice MMR vaccinated cases. We used Firth logistic regression where there were zero cases in subgroup analyses [13]. This produces finite parameter estimates by means of penalised maximum likelihood estimation. We adjusted for the age group (⩽13 months, 14 months–8 years, 9–18 years, ⩾19 years) in all analyses. These age-groups were chosen since in the Netherlands children receive MMR1 at 14 months of age and MMR2 at 9 years of age and to distinguish adolescents and adults. Associations with a *P* value below 0.05 were considered statistically significant. We calculated the vaccine effectiveness (VE) for protection against complications/hospitalisation and infectiousness for one and two doses of MMR as VE = 1-aOR. For two doses of MMR, we also estimated the total VE against measles and infectiousness and against measles and complications/hospitalisation as:

VE_Total_ = 1 − ((1 − VE) × (1 − VE_I,C_)), where VE is the VE against measles (which we assumed was 0.94) and VE_I,C_ is the VE against infectiousness or complications (as estimated in our study), whereby both VEs are expressed as fractions rather than percentages [14, 15]. We used STATA software version 14.0 and R for the analyses.

## Results

In total, 2700 measles cases were notified during the 2013–2014 outbreak. Of these, 888 (32.9%) were confirmed by laboratory testing. Twenty-four cases were excluded from the analyses because of unknown vaccination status (*n* = 20) or since their vaccination status was not verified by national vaccination register, by a vaccination card or by a GP (*n* = 4). The median age of cases was 10 years (range 0–68) and 50% were female. Most cases (2,161, 81%) were orthodox Protestants (Table 1). Of 2676 notified cases with known vaccination status, 2539 (94.9%) were unvaccinated, 121 (4.5%) were vaccinated once, 15 (0.6%) were vaccinated twice and one case received three doses. The MHSs verified cases' vaccination status in the national vaccination register (67%), with the vaccination card (24%) or by a GP (9%).

**Table 1.** Characteristics of measles cases by MMR vaccination status, the Netherlands, May 2013–March 2014 (*n* = 2676)

		Number of cases^a^	Unvaccinated *n* (%)^a^	Vaccinated: 1 dose *n* (%)^a^	Vaccinated: 2 doses *n* (%)^a^	Vaccinated: 3 doses *n* (%)^a^	*P* value
*n*^b^		2676	2539 (94.9)	121 (4.5)	15 (0.6)	1 (0.04)	
Median age in years (range)		10 (0–68)	10 (0–68)	5 (0–41)	26 (12–35)	30	
Age group	Infant (⩽13 months)	78 (2.9)	75 (2.9)	3 (2.5)	0 (0)	0 (0)	
	Child (14 months–8 years)	1081 (40.4)	996 (39.2)	85 (70.3)	0 (0)	0 (0)	
	Adolescent (9–18 years)	1292 (48.3)	1275 (50.2)	14 (11.6)	3 (20)	0 (0)	
	Adult (⩾19 years)	225 (8.4)	193 (7.6)	19 (15.7)	12 (80)	1 (100)	<0.000
Sex	Male	1317 (49.2)	1251 (49.3)	59 (48.8)	6 (40)	1 (100)	
	Female	1343 (50.2)	1272 (50.1)	62 (51.2)	9 (60)	0 (0)	
	Unknown	16 (0.6)	16 (0.6)	0 (0)	0 (0)	0 (0)	0.871
Case definition	Confirmed	871 (32.6)	771 (30.4)	87 (71.9)	12 (80)	1 (100)	
	Probable	1805 (67.4)	1768 (69.6)	34 (28.1)	3 (20)	0 (0)	<0.000
Risk group	None	191 (7.1)	108 (4.3)	71 (58.7)	11 (73.4)	1 (100)	
	Orthodox Protestant denomination	2161 (80.8)	2135 (84.1)	24 (19.8)	2 (13.3)	0 (0)	
	Anthroposophist	16 (0.6)	16 (0.6)	0 (0)	0 (0)	0 (0)	
	Critical attitude towards vaccination	177 (6.6)	172 (6.8)	5 (4.1)	0 (0)	0 (0)	
	Unknown	131 (4.9)	108 (4.2)	21 (17.4)	2 (13.3)	0 (0)	<0.000

a Percentages displayed of column total.

b Percentages displayed of row total.

### Severity

Of 2676 cases with verified vaccination status, the occurrence of complications was known for 2563 (96%). For 328 (13%) of these, complications were reported. Of cases with complications, 311 (95%) reported one complication and 17 (5%) two complications. In total 158 (6%) cases had pneumonia, 113 (4%) otitis media and two (0.1%) cases had encephalitis. Other complications were reported for 72 (3%) cases. These other complications were most often a respiratory infection or dehydration. For 317 (15%) of unvaccinated cases and 11 (10%) of vaccinated cases, a complication was reported (Table 2). All complications, except otitis media, were more prevalent in the unvaccinated group (Table 2). One unvaccinated case with encephalitis and pneumonia died (case fatality ratio among unvaccinated cases 0.04%).

**Table 2.** Measles complications by MMR vaccination status, the Netherlands, May 2013–March 2014 (*n* = 2563)

MMR doses received	Number of cases	Cases with pneumonia *n* (%)	Cases with otitis media *n* (%)	Cases with other complications *n* (%)	Cases with encephalitis *n* (%)	Hospitalised cases *n* (%)^a^
0	2428	153 (6.3)	107 (4.4)	71 (2.9)	2 (0.1)	163 (6.7)
1	119	5 (4.2)	6 (5)	1 (0.8)	0 (0)	9 (7.6)
2^b^	16	0 (0)	0 (0)	0 (0)	0 (0)	0 (0)

a 127 of the unvaccinated hospitalised cases had (a) complication(s) and 2 of the once vaccinated hospitalised cases had (a) complication(s).

b One case received three MMR doses.

In total 172 (7%) cases were hospitalised. The median duration of hospital admission was 4 days and it did not differ between unvaccinated and vaccinated hospitalised cases. Cases of orthodox Protestant denomination (6%) and other risk groups (2%) were less often hospitalied than cases that did not belong to a risk group (14%) (*P* < 0.000) (adjusted for vaccination status).

We combined hospitalisation and complications in the analyses of severity and MMR vaccination status. Of the 2563 cases, 371 (14%) had complications and/or were hospitalised.

Of 2428 unvaccinated cases, 353 (14.5%) had complications and/or were hospitalised and 18 (13.3%) of the 135 vaccinated cases had complications and/or were hospitalised (aOR 0.72 (95% CI 0.5–1.5), p 0.22). Taking into account the number of doses of MMR, 18 (15.1%) of the 119 once-vaccinated cases and none (0%) of the 16 at least twice-vaccinated cases had complications and/or were hospitalised (aOR 0.87 (95% CI 0.5–1.4), *P* = 0.60) and (aOR 0.12 (95% CI 0.0–0.89), *P* = 0.03, VE = 88%), respectively (Table 3). The estimated total VE against measles and against complications/hospitalisation, for two doses of MMR, was 99% (95% CI 11–100).

**Table 3.** Association between severity (any complication and/or hospitalisation) and MMR vaccination status, the Netherlands, May 2013–March 2014

MMR doses received	No. of measles cases	No. (%) of cases with complications	OR (95% CI)	*P* value	VE %	aOR (95% CI)^a^	*P* value^a^	aVE %^a^
0	2428	353 (14.5)	ref	ref	ref	ref	ref	ref
⩾1	135	18 (13.3)	0.90 (0.5–1.5)	0.69	10 (−50–50)	0.72 (0.5–1.5)	0.22	28 (−50–50)
***1***	*119*	*18 (15.1)*	*1.1 (0.6–1.7)*	*0.37*	*−9 (−74–38)*	*0.87* (0.5–1.4)	*0.60*	*13 (−43–49)*
***2***^b^	*16*	*0 (0)*	*0.18 (0–1.3)*	*0.11*	*82 (−32–100)*	*0.12 (0–0.89)*	*0.03*	*88 (11–100)*

a Adjusted for age group (⩽13 months, 14 months–8 years, 9–18 years, ⩾19 years).

b One case received three MMR doses.

### Infectiousness

A total of 709 cases (26%) indicated a source of infection. After correction for the contact period and generation interval, as described in the methods, 376 cases could be linked to 201 likely sources. The mean number of cases linked to a likely source was 1.9, SD 1.35 (range 1–11).

Of 2538 unvaccinated cases, 194 (8%) were reported as a likely source whilst of the 137 vaccinated cases seven (5%) were reported as a likely source (aOR 0.74 (95% CI 0.3–1.6), *P* = 0.45). All vaccinated likely sources had only one secondary case whilst unvaccinated likely sources had a mean of 1.9 secondary cases (*P* = 0.02). Of the seven vaccinated likely sources 71% of their secondary cases were also vaccinated whilst of the 194 unvaccinated likely sources only 5% of their secondary cases were vaccinated.

Taking into account the number of doses of MMR, seven (6%) of the once-vaccinated cases and none (0%) of the 16 at least twice-vaccinated cases were indicated as a likely source (aOR 0.9 (95% CI 0.4–1.8), *P* = 0.77) and (aOR 0.39 (95% CI 0–3), *P* = 0.45, VE = 61%), respectively (Table 4).

**Table 4.** Association between infectiousness and MMR vaccination status, the Netherlands, May 2013–March 2014

MMR doses received	No. of measles cases	No. (%) of cases indicated as a likely source	OR (95% CI)	*P* value	VE %	aOR (95% CI)^a^	*P* value^a^	aVE %^a^
***0***	2538	194 (7.6)	ref	ref	ref	ref	ref	ref
⩾***1***	137	7 (5.1)	0.65 (0.3–1.4)	0.27	35 (−40–70)	0.74 (0.3–1.6)	0.45	26 (−60–70)
***1***	*121*	*7 (5.8)*	*0.79 (0.3–1.6)*	*0.53*	*21 (−57–66)*	*0.90 (0.38–1.80)*	*0.77*	*10 (−80–62)*
***2***^b^	*16*	*0 (0)*	*0.39 (0–2.7)*	*0.56*	*63 (−171–100)*	*0.39 (0–3.0)*	*0.45*	*61 (−203–100)*

a Adjusted for age group (⩽13 months, 14 months–8 years, 9–18 years, ⩾19 years).

b One case received three MMR doses.

The estimated total VE against measles and infectiousness, for two doses of MMR, was 98% (95% CI −203 to 100).

## Discussion

During a measles outbreak in the Netherlands in 2013–2014, we found that none of the at least twice-vaccinated measles cases had complications, neither was hospitalised nor was indicated as a likely source for other cases. Among measles cases, those who were vaccinated with two doses of MMR were less likely to develop complications and/or were hospitalised as a result of measles.

Our results are consistent with findings by others. Misra *et al*., report a lower proportion of complications, such as pneumonia, ear infection, and diarrhoea among at least once-vaccinated cases [16]. In a study of Mitchell *et al*., unvaccinated cases were 2.8 times more likely to have more severe clinical outcomes, such as height and duration of fever, number of days needing medication (other than paracetamol) and days required in bed, compared to vaccinated cases [17]. De Serres *et al*., also found that twice-vaccinated cases had milder illness than those who were unvaccinated or once-vaccinated cases [18]. This is in line with our results, where none of the at least twice-vaccinated cases reported complications. The once-vaccinated cases reported complications, but the proportion of the different complications was lower, albeit not significantly so, for the once-vaccinated cases compared with unvaccinated cases, except for otitis media. In one study, measles vaccination was found to be associated with lower mortality [19]. The low number of deaths in our study did not allow an assessment of the effect of MMR vaccination on measles mortality among cases.

None of the at least twice-vaccinated cases were hospitalised in our study. De Serres *et al*. also showed that twice-vaccinated cases had a significantly lower risk of hospitalisation than those who were unvaccinated or once-vaccinated [18]. Another study also reported lower hospital rates in once-vaccinated cases [20]. In our study, there was no difference between the unvaccinated and once-vaccinated cases, but the reason for hospital admission seems less severe in the vaccinated cases. Among hospitalised vaccinated cases, 30% reported a complication *vs.* 75% of the hospitalised unvaccinated cases. A reason for this can be that unvaccinated cases, most often of Orthodox Reformed denomination are familiar with measles infection in their (large) families and do not seek medical care as often as those who are vaccinated. This is supported by the lower hospitalisation rate among the orthodox reformed risk group compared to cases that do not belong to a risk group. A second reason can be that about 10% of the group of unvaccinated orthodox reformed are not insured and have to pay the hospital admission themselves [10].

In our study, none of the at least twice-vaccinated cases was indicated as a likely source by other cases. A few case reports were published which document the absence of transmission from vaccinated cases [6, 7, 21]. One study described transmission from a twice-vaccinated individual with documented secondary vaccine failure [5]. We found seven once-vaccinated cases who were a likely source to other cases. Of these vaccinated likely sources, three were hospitalised and one had pneumonia. Their relatively severe course of illness and infectiousness may indicate primary vaccine failure. Coleman *et al*. suggested vaccinated cases are less infectious because of the relatively mild nature of their illness [22].

The relatively small proportion of vaccinated cases during this outbreak, compared with other outbreaks in Europe [23–27], limited the power of our analyses. Another limitation is that we could not distinguish the role of primary or secondary vaccine failure since we lacked information on the immune response and avidity levels [28] of vaccinated cases.

During this outbreak, only 9% of measles cases were notified [29], consistent with the underreporting estimated in the previous outbreak [30, 31]. The proportion of complications and hospitalisations among all infected individuals might be lower than the proportion among notified cases when taking the underreporting into account. Cases with complications and hospitalised cases will probably be notified, because of the severity of the disease. In the recent underreporting study, the proportion of unreported cases in the vaccinated group was 88% and in the unvaccinated group 91%. However, we believe that underreporting of cases did not bias our results since we focussed on relative severity and infectiousness of vaccinated compared to unvaccinated cases rather than absolute severity and infectiousness.

It is possible that cases developed complications after being notified, thus leading to underestimation of the frequency of complications. However, we do not believe there is a relation between the completeness of reporting complications and the vaccination status of cases.

For only a small percentage of the cases, we identified a likely source (14%), caused by the underreporting of cases and the strict definition for transmission we applied. In vaccinated cases, the source of infection was easier to identify for cases that do not belong to a risk group than in the group of orthodox Protestant denomination, because there were many orthodox Protestant cases.

Besides the results could be biased because vaccinated cases mainly have contact with vaccinated cases and unvaccinated cases with unvaccinated. Therefore, the calculated VEi can be overestimated. We tried to analyse this by assessing the vaccination status of the secondary cases of the likely sources. The results show that vaccinated cases indeed tend to cluster with vaccinated cases and unvaccinated with unvaccinated cases. As vaccinated cases have less chance to get measles infection, the probability of transmission of measles to vaccinated individuals is lower than the probability of transmission to unvaccinated individuals. This bias can lead to underestimation of the OR and overestimation of the VE. We intended to carry out the analyses on the transmission for the risk group of orthodox Protestant denomination only because this (mainly unvaccinated) group tends to cluster. Unfortunately, there were no vaccinated likely sources in this group and therefore we could not assess the presence of this bias.

In conclusion, our findings suggest a protective effect of MMR vaccination on the occurrence of complications and/or hospitalisation. These are important findings for global measles control policies. None of the at least twice-vaccinated cases had complications, were hospitalised or were indicated as a likely source to other cases. Our study, therefore, supports the WHO recommendation of a two-dose MMR vaccination schedule [2]. The severity and infectiousness of vaccinated measles cases are important indicators for measles surveillance and outbreak investigation. We recommend measles surveillance including these indicators.
